# List of Accidents Admitted into the Pennsylvania Hospital, from February 7th to February 14th, 1838

**Published:** 1838-02-28

**Authors:** 


### PENNSYLVANIA HOSPITAL.

*List of Accidents admitted into the Pennsylvania Hospital, from February 7th to February 14th*, 1838.

One contusion of the hip, discharged four days after at his own request, one bite of a dog, one fracture of the tibia and fibula, and one of the acromion scapulæ in a female.

The fracture of the radius, in a boy, mentioned in No. 3, was discharged, cured in 20 days. The fractured rib, complicated with wound of the lung, admitted on the 14th ultimo, (see No. 3,) was discharged, cured in 32 days. The contusion of the side, admitted on the 23d January, was discharged, cured in 29 days. The case of fractured fibulæ admitted on the 18th January, was discharged, cured in 34 days.

